# Therapeutic Role of Curcumin in Diabetes: An Analysis Based on Bioinformatic Findings

**DOI:** 10.3390/nu14153244

**Published:** 2022-08-08

**Authors:** Ali Mahmoudi, Stephen L. Atkin, Nikita G. Nikiforov, Amirhossein Sahebkar

**Affiliations:** 1 Department of Medical Biotechnology and Nanotechnology, Faculty of Medicine, Mashhad University of Medical Sciences, Mashhad, Iran; 2 School of Postgraduate Studies and Research, RCSI Medical University of Bahrain, Busaiteen 15503, Bahrain; 3 Laboratory of Angiopathology, Institute of General Pathology and Pathophysiology, 125315 Moscow, Russia; 4 Applied Biomedical Research Center, Mashhad University of Medical Sciences, Mashhad, Iran; 5 Biotechnology Research Center, Pharmaceutical Technology Institute, Mashhad University of Medical Sciences, Mashhad, Iran; 6 Department of Biotechnology, School of Pharmacy, Mashhad University of Medical Sciences, Mashhad, Iran

**Keywords:** curcumin, diabetes, DGIdb, DisGeNET, gene ontology, KEGG, STITCH

## Abstract

Background: Diabetes is an increasingly prevalent global disease caused by the impairment in insulin production or insulin function. Diabetes in the long term causes both microvascular and macrovascular complications that may result in retinopathy, nephropathy, neuropathy, peripheral arterial disease, atherosclerotic cardiovascular disease, and cerebrovascular disease. Considerable effort has been expended looking at the numerous genes and pathways to explain the mechanisms leading to diabetes-related complications. Curcumin is a traditional medicine with several properties such as being antioxidant, anti-inflammatory, anti-cancer, and anti-microbial, which may have utility for treating diabetes complications. This study, based on the system biology approach, aimed to investigate the effect of curcumin on critical genes and pathways related to diabetes. Methods: We first searched interactions of curcumin in three different databases, including STITCH, TTD, and DGIdb. Subsequently, we investigated the critical curated protein targets for diabetes on the OMIM and DisGeNET databases. To find important clustering groups (MCODE) and critical hub genes in the network of diseases, we created a PPI network for all proteins obtained for diabetes with the aid of a string database and Cytoscape software. Next, we investigated the possible interactions of curcumin on diabetes-related genes using Venn diagrams. Furthermore, the impact of curcumin on the top scores of modular clusters was analysed. Finally, we conducted biological process and pathway enrichment analysis using Gene Ontology (GO) and KEGG based on the enrichR web server. Results: We acquired 417 genes associated with diabetes, and their constructed PPI network contained 298 nodes and 1651 edges. Next, the analysis of centralities in the PPI network indicated 15 genes with the highest centralities. Additionally, MCODE analysis identified three modular clusters, which highest score cluster (MCODE 1) comprises 19 nodes and 92 edges with 10.22 scores. Screening curcumin interactions in the databases identified 158 protein targets. A Venn diagram of genes related to diabetes and the protein targets of curcumin showed 35 shared proteins, which observed that curcumin could strongly interact with ten of the hub genes. Moreover, we demonstrated that curcumin has the highest interaction with MCODE1 among all MCODs. Several significant biological pathways in KEGG enrichment associated with 35 shared included the AGE-RAGE signaling pathway in diabetic complications, HIF-1 signaling pathway, PI3K-Akt signaling pathway, TNF signaling, and JAK-STAT signaling pathway. The biological processes of GO analysis were involved with the cellular response to cytokine stimulus, the cytokine-mediated signaling pathway, positive regulation of intracellular signal transduction and cytokine production in the inflammatory response. Conclusion: Curcumin targeted several important genes involved in diabetes, supporting the previous research suggesting that it may have utility as a therapeutic agent in diabetes.

## 1. Introduction

Diabetes is a prevalent disease that is considered a widespread issue globally. It is known as a set of metabolic illnesses marked by hyperglycemia caused by insulin production or insulin function problems. Based on the International Diabetes Federation (IDF) Atlas, in 2021, it was reported that 27 million people ranging from 20–79 years of age have diabetes. It is expected that this number will increase to 653 million by 2030. Seventy-five percent of people with diabetes are in low- and middle-income countries [1]. Diabetes is linked to long-term harm, malfunction, and failure of several organs, including the eyes, nerves, heart, kidneys, and blood vessels. Several different pathogenic mechanisms cause diabetes. These include varying from the autoimmune destruction of the beta cells of the pancreas resulting in insulin insufficiency to anomalies that result in insulin resistance. Diabetes causes glucose, lipid, and protein metabolism irregularities due to insulin’s ineffective activity on target tissues. Inadequate insulin production and/or decreased tissue responses cause insulin deficiency along the complicated hormone activity pathways at one or more locations [2,3]. Diabetes in the long term causes numerous consequences: retinopathy, nephropathy, peripheral neuropathy, amputations, Charcot joints, autonomic neuropathy, and sexual dysfunction. It also reported that diabetic induvial increased the risk of having peripheral arterial, atherosclerotic cardiovascular, and cerebrovascular disease. These patients are more likely to have hypertension and impaired lipoprotein metabolism [2]. Diabetes is classified into four kinds: type 1, type 2, gestational diabetes, and secondary or other particular forms of diabetes [4]. Diabetes type 1 accounts for around 5% of all diabetes and is caused by an autoimmune attack on pancreatic islet beta cells. Diabetes type 2 is the other primary type of diabetes, accounting for 90–95 percent of all diabetes cases in the United States and globally [5,6]. Insulin resistance and relative insulin insufficiency combine to create it. Gestational diabetes is unique to pregnancy and is a precursor to diabetes type 2. It affects anywhere from 3% to 9% of all pregnancies [5,6]. Various research supported numerous genes and pathways involved in diabetes mechanisms and associated with β-cell dysfunction, insulin resistance, inflammation, oxidative and nitrative stress, hyperglycemic internal environment, autophagy defects, immune receptors, and other factors related to the development of diabetes [7,8,9].

Curcumin is a traditional medicine derived from *Curcuma longa*, and its features have attracted the attention of many researchers around the world [10]. Curcumin has different properties such as antioxidant, anti-inflammation, anti-cancer, and anti-microbial, which are considered for treating various diseases such as diabetes complications [9,11,12,13,14,15,16,17]. Curcumin indicates that it could regulate diverse genes and pathways, including inflammatory cytokines, growth factors and their receptors, enzymes, adhesion molecules, apoptosis-related proteins, and cell cycle proteins. Because of that, it demonstrates a protective and curative function in various diseases such as diabetes [18,19,20,21,22]. Growth evidence referred to the effectiveness of curcumin in suppressing and controlling diabetes. That evidence suggested that the anti-diabetic properties of curcumin might be related to its capacity to inhibit inflammatory processes and oxidative stress. Furthermore, it is reported that curcumin significantly decreases glycated haemoglobin, fasting blood glucose, triglycerides, very low-density lipoprotein (VLDL)-c, LDL-c, total cholesterol, serum C reactive protein, and body mass index [23]. Consequently, curcumin can be regarded as a therapeutic agent for diabetic patients.

In a recent investigation, virtual screening based on a bioinformatics method has played an important role in the deconvolution and examination of the relationship of medications and diseases [24,25,26].

**Figure 1:**
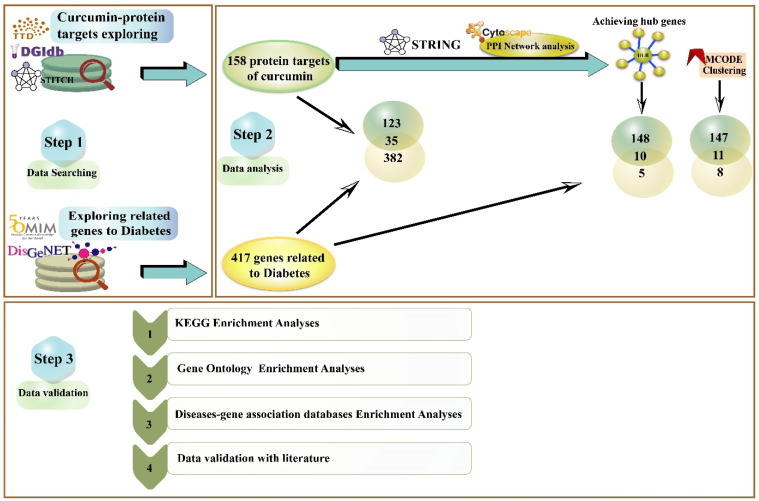
An overview of an investigative process undertaken in the present study. This investigation is organized into three sections: (Step 1) exploring the gene/protein target of curcumin and diabetes in different databases. (Step 2) Analyzing two data sets of curcumin targets and diabetes-related genes/proteins and discovering the associations. (Step 3) Probing the pathways and biological process, and gene-disease enrichment analysis related to obtained important intersection protein/genes, and then validation with a literature review.

With a bioinformatics approach, we aim to investigate the effect of curcumin on critical genes and pathways related to diabetes, which these results might interpret as positive curcumin’s effectiveness in remedying diabetes. In Figure 1, we describe an outlook of our investigation process in our study.

## 2. Methods

### 2.1. Curcumin Targets Exploring

We first searched interactions of curcumin in three different databases, including the STITCH database (http://stitch.embl.de/ accessed on 20 May 2022), therapeutic target database (TTD) (http://db.idrblab.net/ttd/ accessed on 20 May 2022), and drug-gene interaction database (DGIdb) (https://www.dgidb.org/ accessed on 20 May 2022). STITCH is a platform for the diagnosis of interaction between chemicals and proteins. Those databases are a repository for exploring the relation of protein targets with corresponding drugs, and each of them has different information. Here we used all of them to obtain comprehensive information from curcumin targets. Here for the STITCH database, we considered the high confidence cut-off (0.700), and for the two other databases, we discovered all the targets included, and for all databases, we limited species to Homo sapiens.

### 2.2. Exploring Critical Diabetes-Related Genes in OMIM and DisGeNET Databases

Subsequently, we investigated the critical curated protein targets for diabetes on two databases, OMIM (https://www.omim.org/ accessed on 20 May 2022) and the DisGeNET database (https://www.disgenet.org/ accessed on 20 May 2022). DisGeNET is a database that contains a collection of genes associated with specific diseases. Those data are integrated from a variety of sources. We used only curated data that existed in these databases. OMIM is an open-access human database searching for information about genes and their relation to diseases.

### 2.3. Protein-Protein Interaction (PPI) Network

To find important clustering groups and critical genes in the network of diseases, we create a PPI network for all the proteins obtained for diabetes. For this purpose, we first construct a PPI network using a STRING database (https://string-db.org/ accessed on 20 May 2022) with a high confidence score >0.7 and species limited to “Homo sapiens” and then upload the data to Cytoscape (version 3.9.1) to explore Molecular Complex Detection (MCODE) and hub genes (using NetworkAnalyzer (version 4.4.8) plugin on Cytoscape)) in the PPI network based on diabetes-related genes. STRING is a comprehensive website that possesses physical and functional protein-protein interactions. The MCODE plugin of Cytoscape with specifics containing degree cut-off = 4, node score cut-off = 0.2, haircut off, k-core = 2, and maximum depth = 100 was performed. The hub genes were selected based on two critical centralities, including Degree as topological algorithms and Betweenness Centrality as centralities, based on the shortest paths.

### 2.4. Evaluate Curcumin against Protein Targets of Diabetes

In step one of investigating the possible effectiveness of curcumin on protein targets of diabetes, we evaluate the shared protein targets between protein targets of curcumin and gene association with diabetes using Venn diagrams (https://bioinfogp.cnb.csic.es/tools/venny/ accessed on 20 May 2022). In step two, we assay targets of curcumin with the three top scores of the modular clusters analysis of related genes with diabetes in the PPI network. In the last step, we evaluate the effect of curcumin on hub genes obtained from the PPI network based on diabetes-related genes.

### 2.5. Biological Pathways and Process Enrichment Analysis

The biological process and pathway enrichment analysis were accomplished using Gene Ontology (GO) and KEGG based on enrichR (https://maayanlab.cloud/Enrichr/ accessed on 20 May 2022). GO is represented in three complementary levels, including Biological Process, Cellular Component, and Molecular Function. Likewise, KEGG is a repository database that combines genomic, chemical, and systemic functional data. EnrichR is an engine for seeking gene sets beside thousands of annotated gene sets. It integrated information from numerous high-profile projects to supply synthesised data about gene sets [27].

### 2.6. Validation of Shared Genes in Diseases-Genes Databases

We sought and validated shared protein targets in different genes-diseases based on enrichR algorithms (DISEASES, DisGeNET, OMIM Disease, Rare Diseases GeneRIF ARCHS4 Predictions Rare Diseases AutoRIF Gene Lists). 

## 3. Results

### 3.1. Assembly and Analyzing PPI Network 

**Figure 2:**
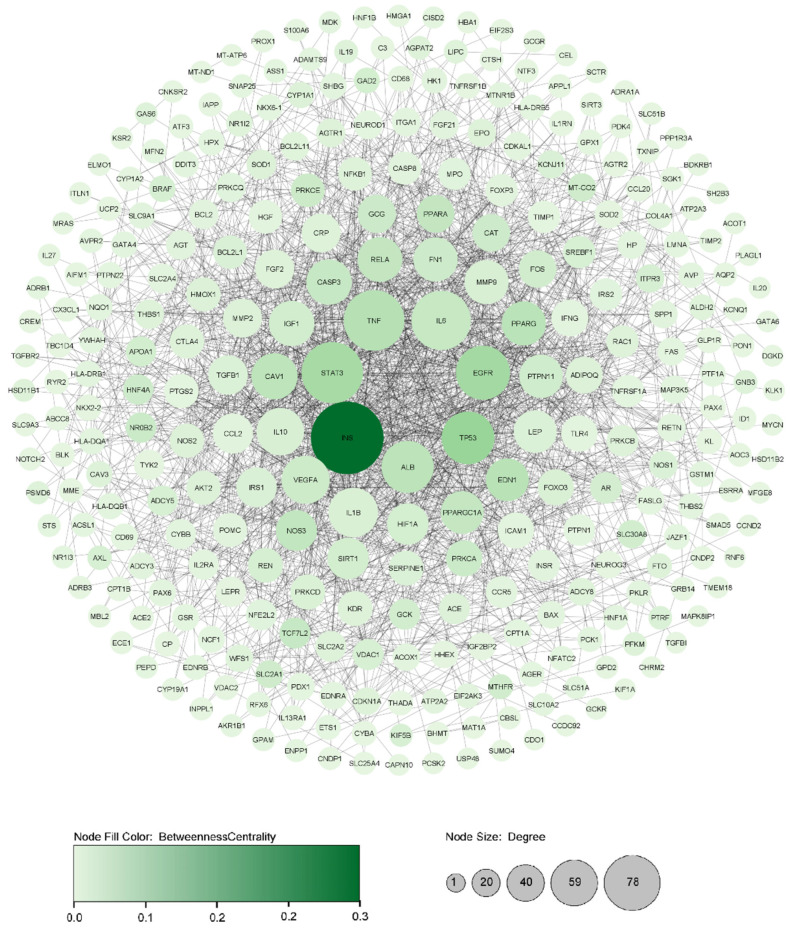
Illustrating critical diabetic disease PPI network based on principal centralities (Degree and Betweenness) using Cytoscape software. The entire PPI network was identified with 298 nodes and 1651 edges. The larger the node size indicates, the higher the Degree, and the higher intensity in node color indicates higher Betweenness in the PPI network.

Based on curated data on DisGeNET and OMIM, 417 genes associated with diabetes were discovered. The constructed PPI network with 417 genes contained 298 nodes and 1651 edges with PPI enrichment *p*-value: <1.0 × 10^−16^ with confidence score >0.7. Next, the analysis of centralities in the PPI network indicated 15 genes with the highest centralities (Betweenness and Degree) (Table 1). We illustrate the Degree with the node’s size and Betweenness with the colour intensifying in Figure 2.

**Figure 3:**
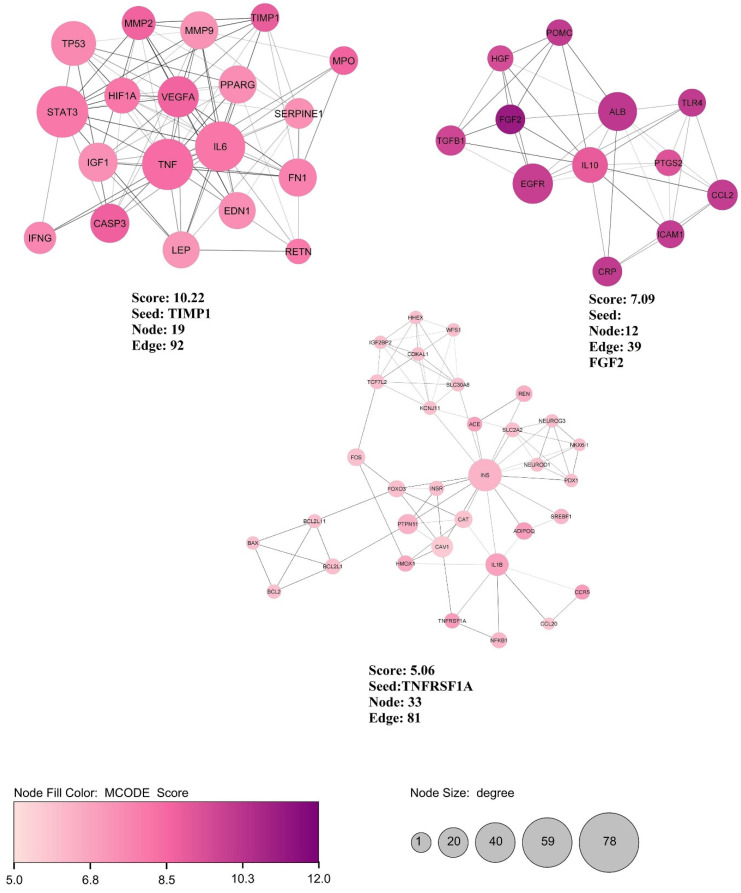
Three top clusters of the PPI network are constructed with important genes related to diabetes based on MCODE analyses. The specification of each MCODE containing MCODE1) Score: 10.22, Seed: TIMP1, Node: 19 Edge: 92. MCODE2) Score: 7.09, Seed: FGF2 Node: 12, Edge: 39. MCODE3) Score: 5.06, Seed: TNFRSF1A Node: 33, Edge: 81. The larger node size indicates a higher degree, and the higher intensity in node color indicates a higher MCODE score in the MCODE analysis.

Besides, MCODE analysis identified three modular clusters based on the cut-off score: 5. The highest score cluster (MCODE 1) comprises 19 nodes and 92 edges with 10.22 scores. TIMP1 has presented the seeds of MCODE 1. Furthermore, FGF2 and TNFRSF1A were the seeds of MCODE 2 and 3, respectively (Figure 3).

### 3.2. Curcumin and their Possible Targets in Diabetes

**Figure 4:**
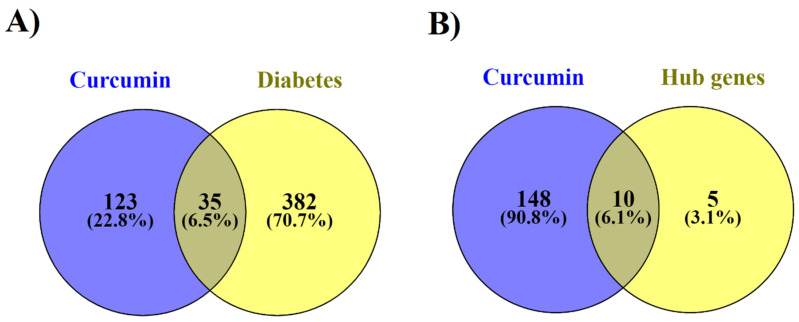
Intersection analysis to determine shared proteins between curcumin and curated genes related to diabetes using the Venn diagram: (**A**) All genes and (**B**) Hub genes.

Screening curcumin interactions in the STITCH (high confidence (0.7)) and DGIdb databases identified 158 protein targets. The Venn diagram of genes related to diabetes and the protein targets of curcumin showed 35 shared proteins. Furthermore, the Venn diagram indicated that ten hub genes of diabetes are significant targets of curcumin (Figure 4). The details of the screening are reported in Table 2.

**Figure 5:**
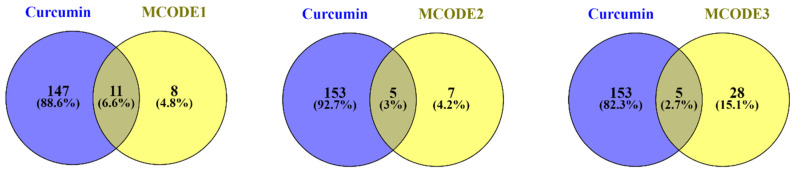
Intersection analysis to determine shared proteins between curcumin and the clusters of PPI network-related genes to diabetes based on MCODEs analysis.

Moreover, we demonstrate that curcumin has the highest interaction with MCODE1 among all MCODs. It could interact with eleven genes, including TP53, CASP3, STAT3, PPARG, MMP9, HIF1A, MMP2, IL6, VEGFA, FN1, and LEP in MCODE1. Curcumin also has interacted with five genes of MCODE2 and MCODE3 (Figure 5).

### 3.3. GO and KEGG Enrichment Analyses of Shared Proteins

**Figure 6:**
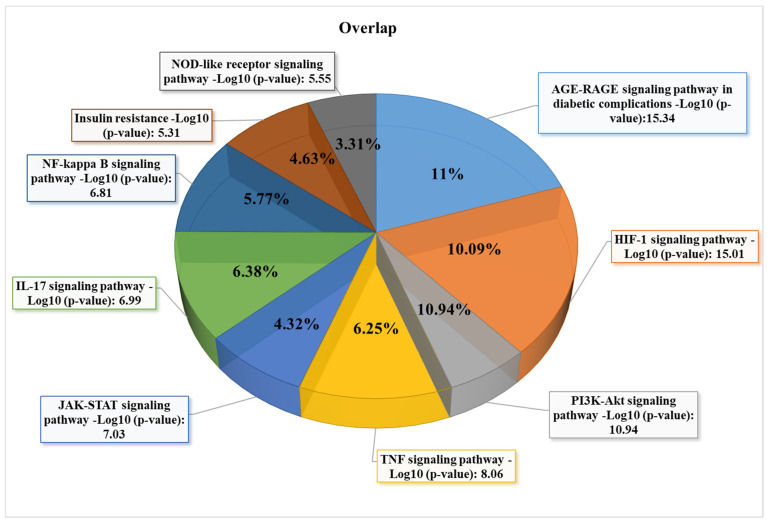
The nine highest adjusted *p*-value signaling pathways were achieved by KEGG enrichment analyses of 35 shared proteins (All genes associated with diabetes ∩ curcumin targets) using the Enrichr algorithm.

We evaluate the biological pathways and processes in the Gene ontology and KEGG databases. Several significant biological pathways associated with 35 shared protein sets were observed in KEGG enrichment. The highest *p*-value pathways included the Advanced glycation end (AGE)-Receptor for Advanced Glycation End (RAGE) signaling pathway in diabetic complications, Hypoxia-inducible Factor (HIF)-1 signaling pathway, Phosphatidylinositol 3-kinase-serine/Threonine Protein Kinase (PI3K-Akt) signaling pathway, Tumor Necrosis Factor (TNF) signaling, and Janus Kinase-signal Transducer And Activator Of Transcription (JAK-STAT) signaling pathway (Figure 6).

**Figure 7:**
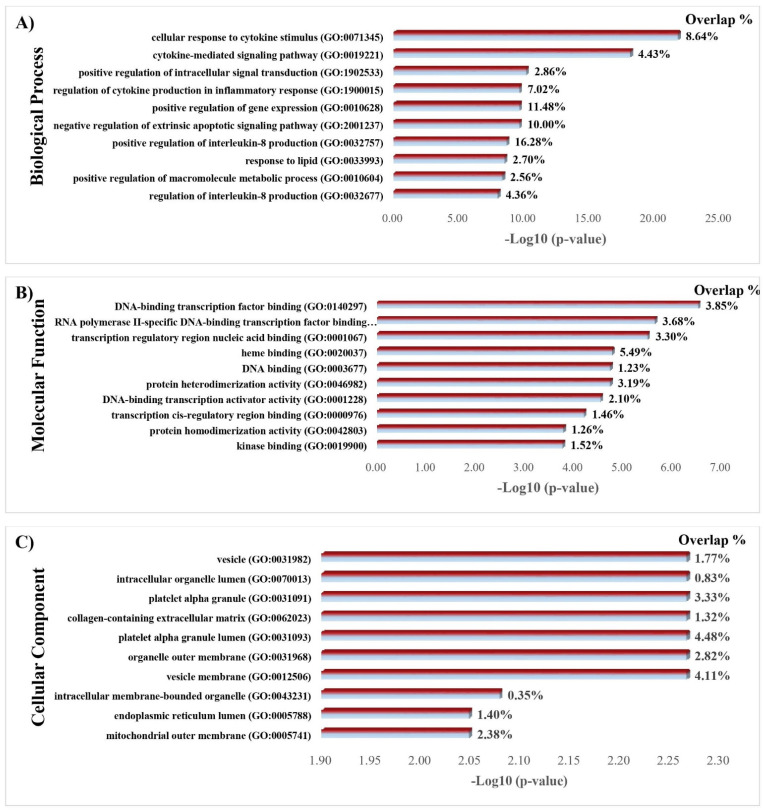
Gene ontology enrichment analysis of 35 shared protein targets (All genes associated with diabetes ∩ curcumin targets) using the Enrichr algorithm. (**A**) Ten highest adjusted *p*-value Biological process; (**B**) Ten highest adjusted *p*-value Cellular compound; (**C**) Ten highest adjusted *p*-value Molecular function.

GO analysis of 35 shared proteins indicated mainly responses to the cellular response to cytokine stimulus, cytokine-mediated signaling pathway, the positive regulation of intracellular signal transduction, the regulation of cytokine production in the inflammatory response, the positive regulation of gene expression, and the negative regulation of the extrinsic apoptotic signaling pathway under biological process. In addition, under molecular function, the analysis showed that these shared proteins were chiefly involved in DNA-binding transcription factor binding, RNA polymerase II-specific DNA-binding transcription factor binding, transcription regulatory region nucleic acid binding, and heme and DNA binding. Moreover, vesicle, intracellular organelle lumen, platelet alpha granule, collagen-containing extracellular matrix, and platelet alpha granule lumen are noticed under cellular components (Figure 7).

### 3.4. Enrichment Analysis of the Different Types of Diabetes in Gene-Diseases Database

Moreover, investigating those 35 shared proteins for diabetes diseases in the various gene-diseases databases, including Jensen DISEASES, DisGeNET, OMIM Disease, Rare Diseases GeneRIF ARCHS4 Predictions Rare Diseases AutoRIF Gene Lists, showed significant relations to different types of diabetes (Table 3). The result indicated that curcumin could impact important genes related to diabetes type 2 and 1, Gestational Diabetes, and other complications and consequences.

## 4. Discussion

Given that different genes are involved in the development and spread of diabetes, by searching and studying these genes, we can target the most important of them and prevent the progression of diseases. Numerous studies have shown that with its antioxidant and anti-inflammatory properties, curcumin can effectively control the disease’s progression. In this bioinformatics study, we investigated the protein targets of curcumin in diabetes along with their biological pathways.

We first sought genes associated with diabetes in the DisGeNET and OMIM databases and selected the curated data. Then, we sought the effective protein interaction of curcumin in two popular drug-gene databases (STITCH and DGIdb). Compared to the two datasets collection, we achieved 35 shared protein targets. Additionally, the biological processes and pathways of 35 shared proteins are suggested that were primarily concerned with inflammatory process and response. The biological pathways that were shown were mainly involved in the AGE-RAGE signaling in diabetic complications, HIF-1, and the PI3K-Akt signaling. By constructing a PPI network from a curated gene collection of diabetes, we reported three top MCODEs and fifteen hub genes. Of fifteen hub genes, ten genes, including TP53, EGFR, STAT3, PPARG, IL6, CASP3, VEGFA, NOS3, PPARA, and FN1, were the targets of curcumin. The results also demonstrated that curcumin is closely associated with three MCODE clusters, especially MCODE1.

In our study, the AGE-RAGE signaling pathway in diabetic complications with a set of genes including IL6, NOS3, CASP3, MMP2, IL1B, STAT3, FN1, BCL2, CCL2, ICAM1, and VEGFA is significantly linked with diabetes and curcumin. In this way, curcumin influencing several genes in this pathway might control diabetes. The AGE-RAGE signaling pathway has been extensively researched in a variety of disease conditions, especially diabetes. The AGE-RAGE signaling cascade has been shown to contribute to increased fibrosis, increased RAGE expression, and higher oxidative stresses [28,29]. By binding to AGE receptors (RAGEs), AGEs modify adaptive and innate immune responses, resulting in reactive oxygen species (ROS), the production of proinflammatory cytokines, and reactive nitrogen intermediates which cause immunosuppression and inflammation. In AGE-related disorders, these pathogenic chemicals affect vascular endothelial/smooth muscular/connective tissue cells and renal mesangial/endothelial/podocyte cells [30]. In hyperglycemic and calcification settings, AGE-RAGE signaling promotes cellular and systemic responses to increase bone matrix proteins via the Protein Kinase C (PKC), fetuin-A, p38 Mitogen-Activated Protein Kinase (MAPK), Nuclear factor kappa-light-chain-enhancer of activated B cells (NF-κB), Transforming Growth Factor-β (TGF- β), and Extracellular Signal-Regulated Kinases ½ (ERK1/2) signaling pathways [28]. Through the activation of Nox-1 and the reduced expression of Superoxide Dismutase-1 (SOD-1), AGE-RAGE signaling has been demonstrated to enhance oxidative stress and accelerate diabetes-related vascular calcification. Increased oxidative stress caused by AGE-RAGE signaling in diabetes-related vascular calcification was also linked to the phenotypic transformation of Vascular Smooth Muscle Cells (VSMCs) to osteoblast-like cells in AGE-induced calcification. According to the researchers, pharmacological treatments and antioxidants were observed to reduce calcium deposition in AGE-induced diabetes-mediated vascular calcification [30]. In a study, transcriptome profile analysis demonstrated that curcumin hindered the AGE-RAGE signaling pathway in diabetes and ameliorated diabetic retinal damage through the antioxidant property [31]. Another study also reported that curcumin neutralizes the effect of AGEs in its function on RAGE and inhibits the activation of hepatic stellate cells (HSC) [32].

Another significant pathway found in enrichment analysis was the HIF1 signaling pathway (Adj-*p*-value: 9.71 × 10^−16^). This pathway, with a set of genes comprising CDKN1A, IL6, NOS2, NOS3, STAT3, BCL2, HMOX1, HIF1A, TLR4, EGFR, and VEGFA, was observed. The activation or inhibition of the HIF-1 signaling pathway indicated involvement with insulin resistance, β-cell dysfunction, and glucose intolerance [33]. It is reported that HIF-1-alpha activates SCOS3 and following it hinders Janus kinase (JAK), which activates STAT3 and hence hinders the expression of adiponectin and promotes insulin resistance [34,35]. The expression amount of HIF-1 in pancreatic β-cells from patients with type 2 diabetes was lowered by 90% compared to non-diabetic control adults. Rodents deficient in HIF-1 expression in β-cells displayed impaired glucose tolerance, decreased insulin production, and abnormal gene expression patterns [36]. Furthermore, HIF1 disruption in β-cells worsens β-cell dysfunction and glucose intolerance by downregulating glycolysis and electron-transport-chain-related gene expression, resulting in lower ATP production [37]. It was also suggested that the Sodium-glucose Cotransporter 2 (SGLT2) inhibitors’ renoprotective effects might be linked to increasing oxygen deprivation signals in the diabetic kidney [38]. According to these findings, hypoxia and HIF signaling may play a crucial part in the pancreatic-cell operation.

The PI3K/Akt pathway is important in our results and was discovered to have a significant relation with curcumin and its targets in diabetic diseases. It was enriched with a set of genes including CDKN1A, IL6, NOS3, FN1, BCL2 FOXO3, THBS1, TP53, TLR4, EGFR, VEGFA, and BCL2L1. In diabetes, the PI3K/Akt signaling pathway has been shown to be involved in all cell processes. It is involved in synthesis, glucose transport, and breakdown and serves as a critical insulin regulator of blood glucose homeostasis. Experiments have shown that upregulating PI3K/Akt activity in diabetes individuals can enhance glucose transporter 4 membrane translocation (GLUT4). This might aid in the decrease in insulin resistance variables and treat gestational diabetes [39]. The PI3K/Akt pathway involved as a factor influencing β-cell volume and function has been demonstrated in vitro and in vivo [40] and is associated with ß-cell dysfunction in type 2 diabetes [41]. Established mice lacking the PI3K/Akt pathway developed severe diabetes and increased ß-cell death [42]. Furthermore, In an STZ (Streptozotocin)-induced diabetes model, a deficit in the PI3K/Akt pathway was characterized [43]. It is crucial to note that transgenic mice overexpressing a constitutively active PI3K/Akt pathway have larger pancreatic β-cells and higher glucose tolerance [44]. A recent study by Ren et al. in 2020 reported that curcumin could provide an active PI3k-Akt signaling pathway and eliminate reactive oxygen species (ROS) in the diabetic rat model [45]. Other recent research suggested that curcumin exerts its anti-diabetic effect chiefly through its anti-apoptotic property and PI3-Akt signaling pathway modulation in the liver [46].

Our study discovered fifteen hub genes from a network analysis of diabetic protein interactions that ten genes indicated are the target of curcumin. Three genes, including STAT3, EGFR, and TP53, were the highest targets in diabetes that strongly curcumin interact with them.

STAT3 (Signal Transducer And Activator Of Transcription 3) activation has been informed to be implicated in the progression of diabetic insulin resistance by regulating set genes involved in glycolipid metabolism and insulin sensitivity [47,48]. In the STZ-induced diabetes animal model and the in vitro high glucose (HG)-stimulated renal tubular epithelial cells, the activation of STAT3 was found. They also reported that the STAT3 inhibitor repressed STAT3 activation in both experimental models, and reduced diabetic nephropathy was observed [49]. The previous studies indicated that metformin and bromocriptine might alleviate hepatic insulin resistance through modulating the STAT3-dependent pathway, insulin sensitivity, and gluconeogenesis [50,51]. This research demonstrates the critical role of the therapeutic action of drugs in regulating STAT3 in diabetic diseases. Numerous studies showed that curcumin exerts its antitumor, chemo-preventive, anti-angiogenesis, and anti-cancer activity via hindering STAT3 phosphorylation and blocking related genes in the STAT3-mediated signaling pathway [52,53,54,55,56]. Previously, it also indicated that curcumin could reduce glomerular sclerosis and albuminuria in diabetic mice models by blocking the phosphorylation of STAT3 [57]. STAT3 in our study was recognized as a key protein interaction based on PPI network analysis with a high score GDA that introduced this gene as one of the critical therapeutic targets for diabetes. It also illustrated that curcumin strongly could inhibit STAT3 (STITCH score: 0.959).

EGFR (epidermal growth factor receptor) is a transmembrane tyrosine kinase receptor belonging to the Erythroblastosis Oncogene B (ErbB) family presented on different mesenchymal, epithelial, and neuronal cells [58]. A study in 2009 reported that in the diabetic rat model, the level of the expression of EGFR was reduced, and an induction in the Ras/Raf/MPK signaling pathway through the increased activation of the signaling elements such as insulin receptor substrate-1 (IRS1) was observed [59]. Another previous study indicated that EGFR has a critical role in progressing STZ-induced diabetes in rat models. In a recent clinical study in a Japanese population with type 2 diabetes, soluble EGFR as a hepatokine was indicated to be associated with insulin resistance in the liver [60]. In clinical research in 2020, over 7.6 years of monitoring and follow-up reported that the change of the annual mean EGFR in type 1 diabetes was −5.7 and in healthy people, it was 0.6 mL/min/1.73 m^2^, which demonstrates the potential rapid biomarker and possible therapeutic targets for individuals with type 2 diabetes [61]. Other previous studies indicated that EGFR has a critical role in the progression of STZ-induced diabetes in rat models [62]. It also demonstrated that curcumin inhibits hyperglycemia and the invasion and migration of pancreatic cancer cells through suppressing the EGF/EGFR signaling pathway and its downstream-related pathways like Akt and ERK [63]. Other research showed that a concentration-dependent dose reduces EGFR phosphorylation by inducing EGFR degradation and suppressing cell proliferation in various gefitinib-resistant non-small-cell lung carcinoma cell lines [64]. Likewise, numerous studies referred to curcumin inhibition and regulating EGFR in different diseases [64]. In our analysis, EGFR showed great impact in the PPI network of diabetes and scored a GDA of 0.37, which curcumin strongly interacts with, and it blocks its function with the highest score among other curcumin targets based on the STITCH database (STITCH score: 0.987).

TP53 (Tumor protein p53) is considered the security of the genome with primary activity as a tumour suppressor. It regulates a vast of signaling pathways related to suppressing oncogenic transformation [65]. Various investigations have referred to the influence of TP53 on diabetes. For example, in a high-fat diet animal, the metabolic stress causes the activation of TP53, which induces cell senescence and insulin resistance. However, many studies have reported conflicting results in relation to diabetes. Some studies have shown that activating it causes diabetes, while others have reported it stopping diabetes [66,67,68,69]. A polymorphisms study of the Chinese population in the case-control form design in 2011 indicated that TP53 is significantly associated with the risk of type 2 diabetes [70]. Furthermore, a study on the European population also indicates that TP53 is linked with the prevalence of Type 2 diabetes (T2D) [71]. Other polymorphism investigations in the form of the cross-sectional study indicated that TP53 impacts insulin resistance in patients with type 2 diabetes independently of body mass [72]. On the other hand, it was reported that curcumin might, through hindering the expression of microRNA-125a-5p, increase the expression of TP53 [73]. Other studies also showed that after curcumin administration, the levels of the TP53 were significantly elevated in rat cells with oxidative DNA impairment [74]. Curcumin stimulates the protein interaction of NAD(P)H Quinone Dehydrogenase 1 (NQO1) with TP53. Consequently, it raises the half-life of TP53 and enables the cytotoxic impact of curcumin [75]. Our obtained data shows that TP53 is closely related to diabetes and is considered one of the most critical hub genes in diabetes. This gene showed that it is strongly regulated with curcumin (STITCH: 0.962).

## 5. Conclusions

In summary, we explored critical diabetes-related genes and pathways that curcumin potentially could interact with. Based on the first obtained result, 35 genes responsible for debates are firmly the target of curcumin. Moreover, our analysis identified 15 vital hub genes involved in the progress and forming of diabetes, and that curcumin strongly regulates 10 genes of these 15 genes. Further study also indicated that curcumin is closely associated with three MCODE clusters, especially MCODE1. According to our results, this interaction and association of curcumin with the related genes with diabetes were in line with ameliorating diabetes based on the literature. We believe that further clinical trials researching will better show the positive effect of curcumin on the therapeutic of diabetes.

## Figures and Tables

**Table 1:** Important hub genes associated with diabetes.

Gene Symbol	Gene Full Name	Protein Class	DSI g	Score GDA	Network Analyser		
					Degree	Betweenness	Closeness
INS	Insulin	Plasma proteins	0.445	0.70	78	0.2223	0.5201
TP53	Tumour protein p53	Transcription factors	0.236	0.50	48	0.0746	0.4714
EGFR	Epidermal growth factor receptor	Enzymes	0.295	0.37	50	0.0632	0.4669
STAT3	Signal transducer and activator of transcription 3	Transcription factors	0.320	0.35	61	0.0604	0.4752
TNF	Tumour necrosis factor	Plasma proteins	0.231	0.50	60	0.0451	0.4845
PPARG	Peroxisome proliferator-activated receptor gamma	Nuclear receptors	0.358	0.50	35	0.0411	0.4506
ALB	Albumin	Plasma proteins	0.317	0.60	47	0.0406	0.4655
CAV1	Caveolin 1	Transporters	0.388	0.50	38	0.0387	0.4534
RELA	RELA proto-oncogene, NF-kB subunit	Transcription factors	0.406	0.50	36	0.0338	0.4291
IL6	Interleukin 6	Plasma proteins	0.248	0.50	58	0.0302	0.4744
CASP3	Caspase 3	Enzymes	0.351	0.50	37	0.0295	0.4439
VEGFA	Vascular endothelial growth factor A	Plasma proteins	0.266	0.50	41	0.0237	0.4562
NOS3	Nitric oxide synthase 3	Enzymes	0.378	0.40	30	0.0349	0.4400
PPARA	Peroxisome proliferator activated receptor alpha	Nuclear receptors	0.432	0.30	25	0.0337	0.4267
FN1	Fibronectin 1	Plasma proteins	0.365	0.40	35	0.0233	0.4273

**Table 2:** The hub genes of the diabetes PPI network that are the targets of curcumin.

Shared Protein Targets	STITCH-Score	Action
TP53	0.962	Activation/inhibition
EGFR	0.987	inhibition
STAT3	0.959	inhibition
PPARG	0.957	Activation
IL6	0.869	inhibition
CASP3	0.959	Activation/inhibition
VEGFA	0.868	inhibition
NOS3	0.820	Activation/inhibition
PPARA	0.866	Activation
FN1	0.844	inhibition

**Table 3:** Enrichment analysis of 35 shared genes through diverse gene association diseases databases based on Enrichr algorithm for diabetic diseases.

Jensen Diseases		
Diseases	Adj. *p*-Value	Gene Name
Diabetic retinopathy	8.32 × 10^−8^	IL6; NOS3; AKR1B1; ICAM1; VEGFA
Diabetes mellitus (1,2)	5.67 × 10^−5^	LEP; STAT3; PPARG; SLC2A4
Type 2 diabetes mellitus	0.027	IL1B; PPARG; VEGFA
GWAS Catalog		
Type 2 diabetes	0.01278	LEP; STAT3; PPARG; VEGFA; BCL2
DisGeNET		
Diseases	Adj. *p*-value	Gene Name
Diabetes Mellitus, Non-Insulin-Dependent	2.33 × 10^−31^	CDKN1A; AKR1B1; SLC2A4; PTGS2; HIF1A; EGFR; ICAM1; CASP3; HMOX1; CCL2; GSTM1; NOS2; NOS3; MMP2; STAT3; FN1; MMP9; VEGFA; AR; IL6; IL1B; LEP; DDIT3; CYP1A2; BCL2; CYP1A1; IAPP; PPARG; PPARA; TP53; TLR4; BCL2L1; NFE2L2
Diabetes Mellitus, Insulin-Dependent	1.56 × 10^−27^	AKR1B1; SLC2A4; PTGS2; EGFR; ICAM1; CASP3; HMOX1; CCL2; GSTM1; NOS2; NOS3; STAT3; FN1; MMP9; VEGFA; AR; IL6; IL1B; LEP; DDIT3; BCL2; IAPP; PPARG; TP53; TLR4; BCL2L1
Diabetic Nephropathy	1.73 × 10^−29^	CDKN1A; AKR1B1; PTGS2; HIF1A; THBS1; EGFR; ICAM1; CCL2; GSTM1; NOS2; NOS3; MMP2; STAT3; FN1; MMP9; VEGFA; IL6; IL1B; LEP; BCL2; IAPP; PPARG; PPARA; TLR4; NFE2L2
Diabetic Retinopathy	5.79 × 10^−26^	GSTM1; NOS2; NOS3; MMP2; FN1; AKR1B1; PTGS2; HIF1A; THBS1; MMP9; ICAM1; VEGFA; IL6; CASP3; CCL2; PPARG; PPARA; TLR4; NFE2L2
Gestational Diabetes	2.02 × 10^−12^	AR; IL6; NOS3; IL1B; LEP; CCL2; PPARG; VEGFA
Prediabetes syndrome	5.82 × 10^−9^	IL6; IAPP; PPARG; SLC2A4; TP53; TLR4
Brittle diabetes	1.54 × 10^−4^	NOS3; DDIT3; STAT3
OMIM Disease		
Diseases	Adj. *p*-value	Gene Name
diabetes mellitus, type 2	0.567 × 10^−3^	SLC2A4; PPARG
Rare Diseases GeneRIF ARCHS4 Predictions		
Diseases	Adj. *p*-value	Gene Name
Diabetic mastopathy	5.515 × 10^−6^	STAT3; IL6; IL1B; TLR4; NFE2L2; PTGS2
Rare Diseases AutoRIF Gene Lists		
Diseases	Adj. *p*-value	Gene Name
Insulin-resistance type B	4.197 × 10^−28^	NOS2; NOS3; STAT3; SLC2A4; PTGS2; EGFR; ICAM1; IL6; CASP3; IL1B; LEP; HMOX1; CCL2; IAPP; PPARG; TLR4; NFE2L2
Diabetic mastopathy	1.58 × 10^−22^	CDKN1A; STAT3; AKR1B1; FOXO3; PTGS2; HIF1A; THBS1; MMP9; ICAM1; VEGFA; IL6; CASP3; DDIT3; BCL2; CYP1A1; TP53; BCL2L1
Nephrogenic diabetes insipidus	2.45 × 10^−5^	NOS2; NOS3; PTGS2; EGFR; NFE2L2
Cardiomyopathy diabetes deafness	6.12 × 10^−4^	NOS3; HMOX1
Maturity-onset diabetes of the young	9.82 × 10^−4^	CASP3; IAPP; PPARG
Neurogenic diabetes insipidus	0.006854232	CASP3; PTGS2

## Data Availability

Not applicable.

## Abbreviations

Acronyms	Full Name
IDF	International Diabetes Federation
VLDL	Very low density lipoprotein
TTD	Therapeutic target database
DGIdb	Drug-gene interaction database
OMIM	Online Mendelian Inheritance in Man
DisGeNET	Gene-disease Associations
PPI	Protein-Protein interaction
STRING	Search Tool For The Retrieval Of Interacting Genes
STITCH	Search Tool For Interactions Of Chemicals
MCODE	Molecular Complex Detection
GO	Gene Ontology
KEGG	Kyoto Encyclopedia of Genes and Genomes
HIF-1	Hypoxia-inducible Factor-1
TNF	Tumor Necrosis Factor
JAK-STAT	Janus Kinase-signal Transducer And Activator Of Transcription
ROS	Reactive oxygen species
AGE-RAGE	Advanced glycation end-Receptor for Advanced Glycation End
PKC	Protein Kinase C
MAPK	Mitogen-Activated Protein Kinase
NF-κB	Nuclear factor kappa-light-chain-enhancer of activated B cells
TGF-β	Transforming Growth Factor-β
ERK1/2	Extracellular Signal-Regulated Kinases 1/2
SOD-1	Superoxide Dismutase-1
VSMCs	Vascular Smooth Muscle Cells
HSC	Hepatic stellate cells
SGLT2	Sodium-glucose Cotransporter 2
GLUT4	Glucose transporter 4 membrane translocation
STZ	Streptozotocin
STAT3	Signal Transducer And Activator Of Transcription 3
HG	High glucose
EGFR	Epidermal growth factor receptor
ErbB	Erythroblastosis Oncogene B
IRS1	Insulin receptor substrate-1
T2D	Type 2 diabetes
NQO1	NAD(P)H Quinone Dehydrogenase 1
INS	Insulin
TP53	Tumor protein p53
EGFR	Epidermal growth factor receptor
PPARG	Peroxisome proliferator activated receptor gamma
ALB	Albumin
CAV1	Caveolin 1
RELA	RELA proto-oncogene, NF-kB subunit
IL6	Interleukin 6
CASP3	Caspase 3
VEGFA	Vascular endothelial growth factor A
NOS3	Nitric oxide synthase 3
PPARA	Peroxisome proliferator activated receptor alpha
FN1	Fibronectin 1
THBS1	Thrombospondin 1
TLR4	toll like receptor 4
FOXO3	forkhead box O3
HMOX1	heme oxygenase 1
ICAM1	intercellular adhesion molecule 1
IL1B	interleukin 1 beta
MMP2	matrix metallopeptidase 2(MMP2)
BCL2	BCL2 apoptosis regulator(
CDKN1A	cyclin dependent kinase inhibitor 1A

## References

[1] Wang H., Li N., Chivese T., Werfalli M., Sun H., Yuen L., Hoegfeldt C.A., Elise Powe C., Immanuel J., Karuranga S. (2022). IDF Diabetes Atlas: Estimation of Global and Regional Gestational Diabetes Mellitus Prevalence for 2021 by International Association of Diabetes in Pregnancy Study Group’s Criteria. Diabetes Res. Clin. Pract..

[2] American Diabetes Association (2014). Diagnosis and classification of diabetes mellitus. Diabetes Care.

[3] Galicia-Garcia U., Benito-Vicente A., Jebari S., Larrea-Sebal A., Siddiqi H., Uribe K.B., Ostolaza H., Martín C. (2020). Patho-physiology of Type 2 Diabetes Mellitus. Int. J. Mol. Sci..

[4] American Diabetes Association (2013). Diagnosis and classification of diabetes mellitus. Diabetes Care.

[5] Genuth S.M., Palmer J.P., Nathan D.M., Cowie C.C., Casagrande S.S., Menke A., Cissell M.A., Eberhardt M.S., Meigs J.B., Gregg E.W., Knowler W.C., Barrett-Connor E., Becker D.J. (2018). Classification and Diagnosis of Diabetes. Diabetes in America.

[6] Centers for Disease Control and Prevention (2014). Prevention. National Diabetes Statistics Report: Estimates of Diabetes and Its Burden in the United States.

[7] Javed Shaikh M.A., Roshan S., Singh H., Rawat S., Pathak S., Mishra A., Gupta G. (2021). Role of Various Gene Expressions in Etiopathogenesis of Type 2 Diabetes Mellitus. Adv. Mind-Body Med..

[8] Marucci A., Rutigliano I., Fini G., Pezzilli S., Menzaghi C., Di Paola R., Trischitta V. (2022). Role of Actionable Genes in Pursuing a True Approach of Precision Medicine in Monogenic Diabetes. Genes.

[9] Miao C., Chen H., Li Y., Guo Y., Xu F., Chen Q., Zhang Y., Hu M., Chen G. (2021). Curcumin and its analog alleviate diabetes-induced damages by regulating inflammation and oxidative stress in brain of diabetic rats. Diabetol. Metab. Syndr..

[10] Sanidad K.Z., Sukamtoh E., Xiao H., McClements D.J., Zhang G. (2019). Curcumin: Recent Advances in the Development of Strategies to Improve Oral Bioavailability. Annu. Rev. Food Sci. Technol..

[11] Mahmoudi A., Kesharwani P., Majeed M., Teng Y., Sahebkar A. (2022). Recent advances in nanogold as a promising nanocarrier for curcumin delivery. Colloids Surf. B Biointerfaces.

[12] Bagherniya M., Nobili V., Blesso C.N., Sahebkar A. (2018). Medicinal plants and bioactive natural compounds in the treatment of non-alcoholic fatty liver disease: A clinical review. Pharmacol. Res..

[13] Farhood B., Mortezaee K., Goradel N.H., Khanlarkhani N., Salehi E., Nashtaei M.S., Najafi M., Sahebkar A. (2019). Curcumin as an anti-inflammatory agent: Implications to radiotherapy and chemotherapy. J. Cell. Physiol..

[14] Shakeri A., Cicero A.F.G., Panahi Y., Mohajeri M., Sahebkar A. (2019). Curcumin: A naturally occurring autophagy modulator. J. Cell. Physiol..

[15] Afshari A.R., Jalili-Nik M., Abbasinezhad-Moud F., Javid H., Karimi M., Mollazadeh H., Jamialahmadi T., Sathyapalan T., Sahebkar A. (2021). Anti-tumor effects of curcuminoids in glioblastoma multiforme: An updated literature review. Curr. Med. Chem..

[16] Gorabi A.M., Kiaie N., Hajighasemi S., Jamialahmadi T., Majeed M., Sahebkar A. (2019). The effect of curcumin on the differentiation of mesenchymal stem cells into mesodermal lineage. Molecules.

[17] Heidari Z., Daei M., Boozari M., Jamialahmadi T., Sahebkar A. (2022). Curcumin supplementation in pediatric patients: A systematic review of current clinical evidence. Phytother. Res..

[18] Shishodia S. (2013). Molecular mechanisms of curcumin action: Gene expression. BioFactors.

[19] Mahmoudi A., Butler A.E., Majeed M., Banach M., Sahebkar A. (2022). Investigation of the Effect of Curcumin on Protein Targets in NAFLD Using Bioinformatic Analysis. Nutrients.

[20] Radbakhsh S., Momtazi-Borojeni A.A., Mahmoudi A., Sarborji M.R., Hatamipour M., Moallem S.A., Atkin S.L., Sahebkar A., Sahebkar A., Sathyapalan T. (2021). Investigation of the Effects of Difluorinated Curcumin on Glycemic Indices in Streptozotocin-Induced Diabetic Rats. Natural Products and Human Diseases: Pharmacology, Molecular Targets, and Therapeutic Benefits.

[21] Panahi Y., Khalili N., Sahebi E., Namazi S., Reiner Ž., Majeed M., Sahbekar A. (2017). Curcuminoids modify lipid profile in type 2 diabetes mellitus: A randomized controlled trial. Complementary Ther. Med..

[22] Parsamanesh N., Moossavi M., Bahrami A., Butler A.E., Sahebkar A. (2018). Therapeutic potential of curcumin in diabetic complications. Pharmacol. Res..

[23] Marton L.T., Pescinini-e-Salzedas L.M., Camargo M.E.C., Barbalho S.M., Haber J.F.D.S., Sinatora R.V., Detregiachi C.R.P., Girio R.J.S., Buchaim D.V., Cincotto dos Santos Bueno P. (2021). The Effects of Curcumin on Diabetes Mellitus: A Systematic Review. Front. Endocrinol..

[24] Mahmoudi A., Butler A.E., Jamialahmadi T., Sahebkar A. (2021). Target Deconvolution of Fenofibrate in Nonalcoholic Fatty Liver Disease Using Bioinformatics Analysis. BioMed Res. Int..

[25] Mahmoudi A., Heydari S., Markina Y.V., Barreto G.E., Sahebkar A. (2022). Role of statins in regulating molecular pathways following traumatic brain injury: A system pharmacology study. Biomed. Pharmacother..

[26] Likić V.A., McConville M.J., Lithgow T., Bacic A. (2010). Systems Biology: The Next Frontier for Bioinformatics. Adv. Bioinform..

[27] Xie Z., Bailey A., Kuleshov M.V., Clarke D.J.B., Evangelista J.E., Jenkins S.L., Lachmann A., Wojciechowicz M.L., Kropiwnicki E., Jagodnik K.M. (2021). Gene Set Knowledge Discovery with Enrichr. Curr. Protoc..

[28] Daffu G., del Pozo C.H., O’Shea K.M., Ananthakrishnan R., Ramasamy R., Schmidt A.M. (2013). Radical roles for RAGE in the pathogenesis of oxidative stress in cardiovascular diseases and beyond. Int. J. Mol. Sci..

[29] Hutchinson K.R., Lord C.K., West T.A., Stewart J.A. (2013). Cardiac fibroblast-dependent extracellular matrix accumulation is associated with diastolic stiffness in type 2 diabetes. PLoS ONE.

[30] Kay A.M., Simpson C.L., Stewart J.A. (2016). The Role of AGE/RAGE Signaling in Diabetes-Mediated Vascular Calcification. J. Diabetes Res..

[31] Xie T., Chen X., Chen W., Huang S., Peng X., Tian L., Wu X., Huang Y. (2021). Curcumin is a Potential Adjuvant to Alleviates Diabetic Retinal Injury via Reducing Oxidative Stress and Maintaining Nrf2 Pathway Homeostasis. Front. Pharmacol..

[32] Tang Y., Chen A. (2014). Curcumin eliminates the effect of advanced glycation end-products (AGEs) on the divergent regulation of gene expression of receptors of AGEs by interrupting leptin signaling. Lab. Investig..

[33] Gonzalez F.J., Xie C., Jiang C. (2018). The role of hypoxia-inducible factors in metabolic diseases. Nat. Rev. Endocrinol..

[34] Jiang C., Kim J.H., Li F., Qu A., Gavrilova O., Shah Y.M., Gonzalez F.J. (2013). Hypoxia-inducible factor 1α regulates a SOCS3-STAT3-adiponectin signal transduction pathway in adipocytes. J. Biol. Chem..

[35] Kanatani Y., Usui I., Ishizuka K., Bukhari A., Fujisaka S., Urakaze M., Haruta T., Kishimoto T., Naka T., Kobayashi M. (2007). Effects of pioglitazone on suppressor of cytokine signaling 3 expression: Potential mechanisms for its effects on insulin sensitivity and adiponectin expression. Diabetes.

[36] Gunton J.E., Kulkarni R.N., Yim S., Okada T., Hawthorne W.J., Tseng Y.H., Roberson R.S., Ricordi C., O’Connell P.J., Gonzalez F.J. (2005). Loss of ARNT/HIF1beta mediates altered gene expression and pancreatic-islet dysfunction in human type 2 diabetes. Cell.

[37] Cheng K., Ho K., Stokes R., Scott C., Lau S.M., Hawthorne W.J., O’Connell P.J., Loudovaris T., Kay T.W., Kulkarni R.N. (2010). Hypoxia-inducible factor-1alpha regulates beta cell function in mouse and human islets. J. Clin. Investig..

[38] Packer M. (2021). Mechanisms Leading to Differential Hypoxia-Inducible Factor Signaling in the Diabetic Kidney: Modulation by SGLT2 Inhibitors and Hypoxia Mimetics. Am. J. Kidney Dis..

[39] Li H.X., Li X.H., Jiang J., Shi P.X., Zhang X.G., Tian M. (2020). Effect of miR-26b on gestational diabetes mellitus in rats via PI3K/Akt signaling pathway. Eur. Rev. Med. Pharmacol. Sci..

[40] Elghazi L., Rachdi L., Weiss A.J., Cras-Méneur C., Bernal-Mizrachi E. (2007). Regulation of beta-cell mass and function by the Akt/protein kinase B signalling pathway. Diabetes Obes. Metab..

[41] Dickson L.M., Rhodes C.J. (2004). Pancreatic beta-cell growth and survival in the onset of type 2 diabetes: A role for protein kinase B in the Akt?. Am. J. Physiol. Endocrinol. Metab..

[42] Bernal-Mizrachi E., Fatrai S., Johnson J.D., Ohsugi M., Otani K., Han Z., Polonsky K.S., Permutt M.A. (2004). Defective insulin secretion and increased susceptibility to experimental diabetes are induced by reduced Akt activity in pancreatic islet beta cells. J. Clin. Investig..

[43] Cui W., Zhang Y., Lu D., Ren M., Yuan G. (2016). Upregulation of p-Akt by glial cell line-derived neurotrophic factor ameliorates cell apoptosis in the hippocampus of rats with streptozotocin-induced diabetic encephalopathy. Mol. Med. Rep..

[44] Bernal-Mizrachi E., Wen W., Stahlhut S., Welling C.M., Permutt M.A. (2001). Islet beta cell expression of constitutively active Akt1/PKB alpha induces striking hypertrophy, hyperplasia, and hyperinsulinemia. J. Clin. Investig..

[45] Ren B.C., Zhang Y.F., Liu S.S., Cheng X.J., Yang X., Cui X.G., Zhao X.R., Zhao H., Hao M.F., Li M.D. (2020). Curcumin alleviates oxidative stress and inhibits apoptosis in diabetic cardiomyopathy via Sirt1-Foxo1 and PI3K-Akt signalling pathways. J. Cell. Mol. Med..

[46] Xia Z.H., Zhang S.Y., Chen Y.S., Li K., Chen W.B., Liu Y.Q. (2020). Curcumin anti-diabetic effect mainly correlates with its anti-apoptotic actions and PI3K/Akt signal pathway regulation in the liver. Food Chem. Toxicol..

[47] Senn J.J., Klover P.J., Nowak I.A., Zimmers T.A., Koniaris L.G., Furlanetto R.W., Mooney R.A. (2003). Suppressor of cytokine signaling-3 (SOCS-3), a potential mediator of interleukin-6-dependent insulin resistance in hepatocytes. J. Biol. Chem..

[48] Mashili F., Chibalin A.V., Krook A., Zierath J.R. (2013). Constitutive STAT3 phosphorylation contributes to skeletal muscle insulin resistance in type 2 diabetes. Diabetes.

[49] Zheng C., Huang L., Luo W., Yu W., Hu X., Guan X., Cai Y., Zou C., Yin H., Xu Z. (2019). Inhibition of STAT3 in tubular epithelial cells prevents kidney fibrosis and nephropathy in STZ-induced diabetic mice. Cell Death Dis..

[50] Kim Y.D., Kim Y.H., Cho Y.M., Kim D.K., Ahn S.W., Lee J.M., Chanda D., Shong M., Lee C.H., Choi H.S. (2012). Metformin ameliorates IL-6-induced hepatic insulin resistance via induction of orphan nuclear receptor small heterodimer partner (SHP) in mouse models. Diabetologia.

[51] Reda E., Hassaneen S., El-Abhar H.S. (2018). Novel Trajectories of Bromocriptine Antidiabetic Action: Leptin-IL-6/ JAK2/p-STAT3/SOCS3, p-IR/p-AKT/GLUT4, PPAR-γ/Adiponectin, Nrf2/PARP-1, and GLP-1. Front. Pharmacol..

[52] Liu Y., Wang X., Zeng S., Zhang X., Zhao J., Zhang X., Chen X., Yang W., Yang Y., Dong Z. (2018). The natural polyphenol curcumin induces apoptosis by suppressing STAT3 signaling in esophageal squamous cell carcinoma 06 Biological Sciences 0601 Biochemistry and Cell Biology 11 Medical and Health Sciences 1112 Oncology and Carcinogenesis. J. Exp. Clin. Cancer Res..

[53] Alexandrow M.G., Song L.J., Altiok S., Gray J., Haura E.B., Kumar N.B. (2012). Curcumin: A novel Stat3 pathway inhibitor for chemoprevention of lung cancer. Eur.J. Cancer Prev..

[54] Khan A.Q., Ahmed E.I., Elareer N., Fathima H., Prabhu K.S., Siveen K.S., Kulinski M., Azizi F., Dermime S., Ahmad A. (2020). Curcumin-mediated apoptotic cell death in papillary thyroid cancer and cancer stem-like cells through targeting of the JAK/STAT3 signaling pathway. Int. J. Mol. Sci..

[55] Liu M., Tian S., Liu Y., Gao F., Yin Q. (2016). Inhibition of curcumin on proliferation and invasion in giant cell tumor of bone (GCTB) by targeting STAT3. Int. J. Clin. Exp. Med..

[56] Xu X., Zhu Y. (2017). Curcumin inhibits human non-small cell lung cancer xenografts by targeting STAT3 pathway. Am. J. Transl. Res..

[57] Lu M., Tao L., Mei W., Luo R., Fu X., Wang L., Yang W., Liu C. (2014). Effect of curcumin on the expression of P-STAT3 and IkB in db/db mice. J. Cent. South Univ. Med. Sci..

[58] Hutchinson R.A., Adams R.A., McArt D.G., Salto-Tellez M., Jasani B., Hamilton P.W. (2015). Epidermal growth factor receptor immunohistochemistry: New opportunities in metastatic colorectal cancer. J. Transl. Med..

[59] Vairaktaris E., Goutzanis L., Yapijakis C., Vassiliou S., Spyridonidou S., Vylliotis A., Nkenke E., Lazaris A.C., Strantzias P., Patsouris E. (2009). Diabetes enhances the expression of H-ras and suppresses the expression of EGFR leading to increased cell proliferation. Histol. Histopathol..

[60] Kyohara M., Shirakawa J., Okuyama T., Togashi Y., Inoue R., Li J., Miyashita D., Terauchi Y. (2020). Soluble EGFR, a hepatokine, and adipsin, an adipokine, are biomarkers correlated with distinct aspects of insulin resistance in type 2 diabetes subjects. Diabetol. Metab. Syndr..

[61] Limonte C.P., Valo E., Montemayor D., Afshinnia F., Ahluwalia T.S., Costacou T., Darshi M., Forsblom C., Hoofnagle A.N., Groop P.H. (2020). A Targeted Multiomics Approach to Identify Biomarkers Associated with Rapid eGFR Decline in Type 1 Diabetes. Am. J. Nephrol..

[62] Akhtar S., Almubrad T., Bron A.J., Yousif M.H.M., Benter I.F., Akhtar S. (2009). Role of epidermal growth factor receptor (EGFR) in corneal remodelling in diabetes. Acta Ophthalmol..

[63] Li W., Wang Z., Xiao X., Han L., Wu Z., Ma Q., Cao L. (2019). Curcumin attenuates hyperglycemia-driven EGF-induced invasive and migratory abilities of pancreatic cancer via suppression of the ERK and AKT pathways. Oncol. Rep..

[64] Lee J.Y., Lee Y.M., Chang G.C., Yu S.L., Hsieh W.Y., Chen J.J.W., Chen H.W., Yang P.C. (2011). Curcumin induces EGFR degradation in lung adenocarcinoma and modulates p38 activation in intestine: The versatile adjuvant for gefitinib therapy. PLoS ONE.

[65] Chakraborty A., Uechi T., Kenmochi N. (2011). Guarding the ’translation apparatus’: Defective ribosome biogenesis and the p53 signaling pathway. Wiley Interdiscip. Rev. RNA.

[66] Wang X., Zhao X., Gao X., Mei Y., Wu M. (2013). A new role of p53 in regulating lipid metabolism. J. Mol. Cell Biol..

[67] Secchiero P., Toffoli B., Melloni E., Agnoletto C., Monasta L., Zauli G. (2013). The MDM2 inhibitor Nutlin-3 attenuates streptozotocin-induced diabetes mellitus and increases serum level of IL-12p40. Acta Diabetol..

[68] Armata H.L., Golebiowski D., Jung D.Y., Ko H.J., Kim J.K., Sluss H.K. (2010). Requirement of the ATM/p53 tumor suppressor pathway for glucose homeostasis. Mol. Cell. Biol..

[69] Franck D., Tracy L., Armata H.L., Delaney C.L., Jung D.Y., Ko H.J., Ong H., Kim J.K., Scrable H., Sluss H.K. (2013). Glucose Tolerance in Mice is Linked to the Dose of the p53 Transactivation Domain. Endocr. Res..

[70] Qu L., He B., Pan Y., Xu Y., Zhu C., Tang Z., Bao Q., Tian F., Wang S. (2011). Association between polymorphisms in RAPGEF1, TP53, NRF1 and type 2 diabetes in Chinese Han population. Diabetes Res. Clin. Pract..

[71] Burgdorf K.S., Grarup N., Justesen J.M., Harder M.N., Witte D.R., Jørgensen T., Sandbæk A., Lauritzen T., Madsbad S., Hansen T. (2011). Studies of the association of Arg72Pro of tumor suppressor protein p53 with type 2 diabetes in a combined analysis of 55,521 Europeans. PLoS ONE.

[72] Bonfigli A.R., Sirolla C., Testa R., Cucchi M., Spazzafumo L., Salvioli S., Ceriello A., Olivieri F., Festa R., Procopio A.D. (2013). The p53 codon 72 (Arg72Pro) polymorphism is associated with the degree of insulin resistance in type 2 diabetic subjects: A cross-sectional study. Acta Diabetol..

[73] Gao W., Chan J.Y., Wong T.S. (2014). Curcumin exerts inhibitory effects on undifferentiated nasopharyngeal carcinoma by inhibiting the expression of miR-125a-5p. Clin. Sci..

[74] Ciftci G., Aksoy A., cenesiz S., sogut M.u., Yarim G.F., Nisbet C., Guvenc D., Ertekin A. (2015). Therapeutic role of curcumin in oxidative DNA damage caused by formaldehyde. Microsc. Res. Tech..

[75] Patiño-Morales C.C., Soto-Reyes E., Arechaga-Ocampo E., Ortiz-Sánchez E., Antonio-Véjar V., Pedraza-Chaverri J., García-Carrancá A. (2020). Curcumin stabilizes p53 by interaction with NAD(P)H:quinone oxidoreductase 1 in tumor-derived cell lines. Redox Biol..

